# Simulation Model for Prediction of Gas Separation in Membrane Contactor Process

**DOI:** 10.3390/membranes12020158

**Published:** 2022-01-28

**Authors:** Choongkyun Yeom, Jiwon Kim, Heeyoung Park, Jiwoong Lee, Seong Eun Park, Boram Gu

**Affiliations:** 1 SepraTek Inc., 730 Gyejok-ro, Daedeok-gu, Daejeon 34396, Korea; jwgim16@sepratek.com (J.K.); nforce5600@sepratek.com (H.P.); jiwlee0509@sepratek.com (J.L.); 2 Envioneer Co., Ltd., 85 Hanbang-expo-ro, Jecheon 27116, Korea; cto@envioneer.com; 3 Chemical Engineering Department, Chonnam National University, 77 Yongbong-ro, Buk-gu, Kwangju 61186, Korea; boram.gu@jnu.ac.kr

**Keywords:** membrane contactor, polypropylene, hollow fiber membrane, gassing, degassing

## Abstract

The purpose of this study is to establish a practical simulation model based on mass balance, mass transport equations and equilibrium equation between gas and liquid phases across a porous membrane in membrane contactor process in order to predict the separation behavior by the gassing process of gas mixture in membrane contactor. The established simulation model was verified by comparison between the simulated values and real process values in the separation of CH_4_/CO_2_ mixture, showing an excellent agreement between them. The parameter R-value in the model, which is a kind of the permeability of permeant across porous membrane, has been determined by fitting a numerical solution of the model equation to the experimental data to obtain a practical value of the parameter. A parametric study on the gassing process of N_2_/CO_2_ mixture in membrane contactor was made with the help of the practical simulation model to investigate the effects of operation parameters on separation performance and to characterize the separation behavior of membrane contactor process. A series of simulations of the separation of N_2_/CO_2_ mixture in membrane contactor were conducted, and the optimization on the membrane process was discussed to maximize the separation performance in terms of N_2_ recovery percent in retentate and CO_2_ permeation rate. It was observed from the analysis of the result of the simulation that liquid flow rate has a negative effect on N_2_ recovery percent in retentate but a positive effect on the separation of CO_2_, while R-value affects the separation performance in the other way. It is confirmed in this study that the developed simulation can be used as a tool to optimize the parameters, i.e., feed gas pressure, liquid flow rate and R-value to maximize the separation performance.

## 1. Introduction

As industrialization rapidly proceeds, water and air become contaminated as fast, so we are facing serious climate change, suffering from a collapsing pattern of regional climate. The demand for clean water and clean air is increasing more and more in environmental, residential, and industrial sectors. A recent emerging technology, called membrane contactor, is gradually used to replace conventional processes in environment, chemical industry, energy, and semiconductor industries, as purification and separation means, for example, replacing the conventional packed tower for producing ultrapure water [1,2,3,4] as well as capturing carbon dioxide from the discharged stream of industrial plants [5,6]. The reason why the membrane contactor receives a lot of attention is that it makes use of a thin permeable membrane in order to increase the overall mass transfer between two fluid phases (e.g., gas/liquid, liquid/liquid), maximizing the mass transfer performance of separation process [7].

Membrane contactors employ microporous hydrophobic hollow fiber membrane, contacting a liquid phase with a gaseous phase by the porous membrane for the preferential mass transfer of gases between the phases [3,4,8]. The hydrophobic membrane employed is not to allow liquid (water) to pass through it into the gaseous phase but to allow gas molecules to freely flow through its porous structure and dissolve into the liquid [9,10]. The membrane contactor process is composed of two processes, i.e., gassing and degassing process, depending on the partial pressure of gas component in the gas stream [1]. When the partial pressure of a gas component is higher than its equilibrium pressure with the concentration of the gas component dissolved in the liquid phase, the gas component is absorbed into the liquid, which is called membrane absorption or gassing process. When the partial pressure of a component in the gas phase goes to the other way, smaller than the equilibrium pressure, the component is desorbed or degassed from the liquid, which is called membrane stripping or degassing process. From this viewpoint, the driving force for the gassing or the degassing process is the pressure difference between partial pressure in the gas phase and the equilibrium pressure with the liquid phase which is expressed as a function of gas concentration in the liquid phase.

Employing a thin permeable membrane and having large mass transfer performance can differentiate the membrane contactor from conventional technologies, such as scrubbing and stripping processes. The attainment of large contact area in membrane contactor process indeed offers unique opportunities for enhanced mass transfer performances as well. Owing to the large contact area and the enhanced mass transfer of membrane contactor, it can require a footprint 5–20 times smaller than conventional separation processes, being in higher compactness, smaller size, and weight to lower capital investment [11,12,13,14].

Membrane contactor performance is dependent mainly on both the membrane module structure design and the process condition. Particularly, liquid flow mode in hollow fiber is of great importance, either lumen-side flow or shell-side flow. The lumen-side flow causes the high pressure drop as well as small membrane area, not favorable for membrane performance. Thus, the shell-side flow is recommended [8,15], rather than the lumen-side flow. The fluid dynamics in the shell-side flow can be affected significantly by module structure design. From the viewpoint of mass-transfer property, the transverse flow of liquid causes a high degree of mixing of feed stream in shell-side flowing of a module and thereby yields high mass transfer, resulting in good membrane performance. It is reported [14,15] that the transverse flow of liquid in the shell side can generate an order higher mass transfer than the lumen-side flow. In the traverse flow of liquid, the streamline of liquid flow is broken by the arrangement of hollow fibers to cause mixing of the liquid stream. 

At our knowledge, a practical simulation model has not been reported to be developed yet to quantitively predict the separation behavior in membrane contactor process. The purpose of this study is to establish a practical simulation model based on mass balance, mass transport equations and equilibrium equation between gas and liquid phases across porous membrane in membrane contactor, to verify the validity of the simulation model by comparison between the simulated values and real process values, and to do a parametric study on the separation characteristics of a gas mixture in membrane contactor process with help of the simulation of the established model. This study focuses on the gassing process of membrane contactor to simplify the establishment of the simulation model. By fitting the model equations to the experimental permeation data of CH_4_/CO_2_ mixture obtained by using the membrane module that was fabricated with the woven mat of porous polypropylene (PP) hollow fiber, a practical value of model parameter, R-value, which has something to do with the mass transfer of permeant, was determined and used to simulate the separation of the gas mixture in membrane contactor process. A parametric investigation on the separation of N_2_/CO_2_ mixture was performed through the simulation of the gassing process, and process optimization was discussed to maximize the separation performance of the membrane process through the parametric study.

## 2. Simulation Model

### 2.1. Simplified Model for Resistance in Membrane Contactor Process

**Figure 1:**
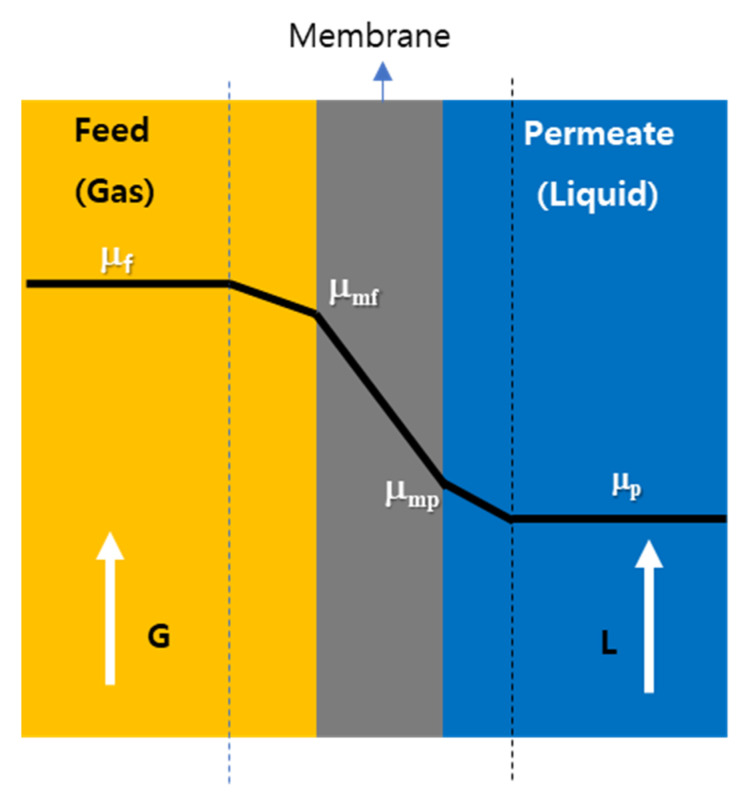
The schematic representation of the chemical potential profile of permeant across membrane system, G = gas flow rate, L = liquid flow rate, μ_f_, μ_mf_, μ_mp_, and μ_p_ chemical potentials of permeant.

A chemical potential gradient rather than a concentration gradient can be taken into consideration as a driving force in a general transport theory to avoid any limits or special cases which are very complicated to make them clear [16,17]. Figure 1 demonstrates the chemical potential profile of permeant across membrane. 

The overall permeation equation of permeant is given in terms of the chemical potential of permeant as follows.

*Jv = K_t_ (μ_f_ − μ_p_)* (1)

where *Jv* is a permeation rate, *K_t_* overall mass transfer coefficient, and *μ_f_* and *μ_p_* the chemical potentials of permeant in feed and permeate, respectively. Three layers, the boundary layer feed, membrane, and the boundary layer in permeate are in series across the membrane system between feed and permeate streams, and the permeation equations across the three layers are described below, respectively.

*Jv = K_bf_ (μ_f_ − μ_mf_)* (2)

*Jv = K_m_ (μ_mf_− μ_mp_)* (3)

*Jv = K_bp_ (μ_mp_− μ_p_)* (4)

The overall mass transfer coefficient *K_t_* can be derived from combining Equations (1)–(4) as shown in Equation (5).

1*/K_t_ =* 1*/K_bf_ +* 1*/K_m_ +* 1*/K_bp_* (5)

which is the equation for resistance(1/*K*) in series connection. 

Usually, in membrane contactor process through a hollow fiber membrane module, liquid flows on transverse mode in the shell side while gas flows in the lumen side of the module to increase mass transfer coefficient [14,15]. In membrane contactor process, the boundary layer resistance in a gaseous feed can be negligible due to very high gas mobility, compared to the membrane resistance through which mass transport can be restricted by its porous structure, as long as the gas flow rate is well higher than permeation rate *Jv, G >> Jv*. In the case of the transverse flow of liquid in shell side of the module with hollow fiber arrangement perpendicular to the liquid flow, the boundary layer resistance in the liquid phase is presumably neglected as well when liquid flows fast enough to create a high degree of mixing of liquid stream flowing through hollow fiber membrane bundle. Thus, the overall resistance will be approximate to the porous membrane resistance (Equation (6)). For large pore size of a porous membrane (Kn << 1, Kn: Knudsen number, defined as the ratio of the mean free path of permeant gas molecule to average pore size), viscous flow or Poiseuille flow occurs in which gas molecules collide exclusively with each other and no separation is obtained between the gaseous components [17].

(6) $$1/K_{t}≅1/K_{m}$$

**Figure 2:**
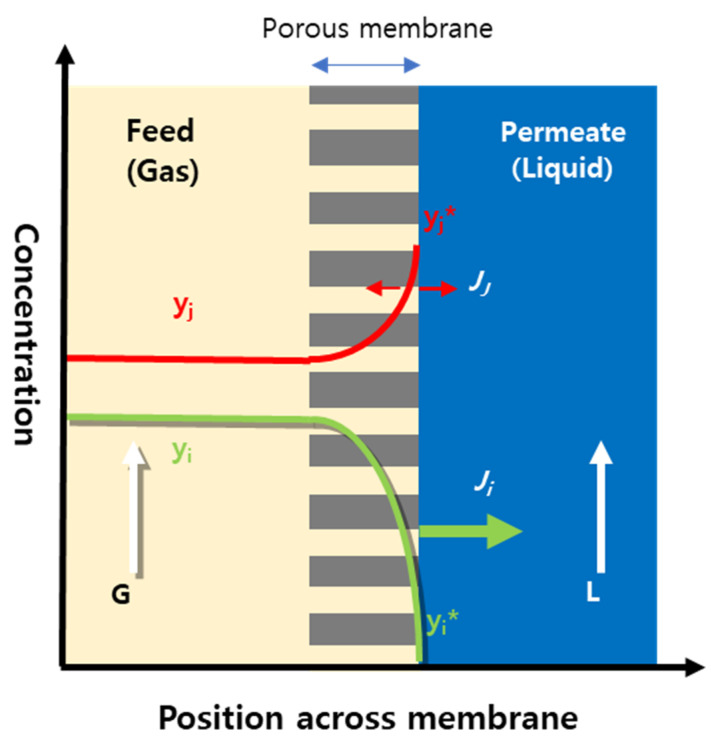
Concentration profile of individual permeant component across porous membrane in membrane contactor: *y_i_* and *y_j_* = concentrations of components *i* (preferentially dissolving component) and *j*, respectively, G and L = gas and liquid flow rates in feed and permeate, respectively, *J_i_* and *J_j_* = fluxes of components *i* and *j*, respectively, and the superscript * denoting gas phase at interface between gas and liquid.

In the gassing process of a gas mixture of components *i/j* (*i*, a selectively dissolving component into the liquid), separation is achieved at the interface by the preferential dissolution of the component *i* to the liquid, so the composition of the feed gas at the interface will be different from the bulk feed stream: lower concentration of component *i* and higher concentration of component *j* at the interface than in the bulk feed. Thus, the resulting concentration profile of individual component can be drawn across the porous membrane as depicted in Figure 2, in taking into consideration the simplified overall resistance model discussed in Equation (6). With permeating through the porous membrane, the component *i* decreases in concentration due to its selective absorption while the component *j* concentration increases correspondingly as the result of more absorbing component *i* to the liquid. Subsequently, the preferential absorption of component *i* will make the dissolution of component *j* to the liquid expedited because the concentration of component *j* increases significantly at the interface as the gaseous feed flows along with membrane module, while the permeation and dissolution of the component *i* can be depressed by further decreasing its concentration at the interface with time due to the depletion of component *i* in feed. Thus, the expedited permeation of the component results in the loss of component *j* into the liquid stream occurring more than expected from the ratio of the solubilities. From this viewpoint, the process optimization should be necessary to minimize the loss via optimizing the concentration profile of individual component across the membrane. 

### 2.2. Model for the Separation of Gas Mixture in the Gassing Process of Membrane Contactor

**Figure 3:**
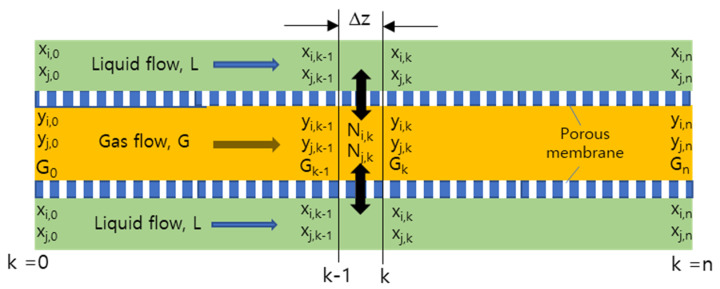
Schematical representation of mass balance across differential section of the hollow fiber membrane module in membrane contactor process: gas flowing in the lumen side of hollow fiber membrane and liquid flowing in the shell side.

In the flow of the two phases through a differential length of volume in membrane module, G, L, x, and y changes slightly (Figure 3). The differential equation showing this is given by the following expression:

Δ*(L x) =*
Δ*(G y)*

This equation is the material balance for over a differential section of the membrane module. The rate at which component *i* or *j* is transferred from one to the other phase through the membrane area in the differential section of the membrane module can be given as below.

Δ*(L x_i_) =*
Δ*(G y_i_) = N_i_ moles/h* (7)

Δ*(Lx_j_) =*
Δ*(G(1 − y_i_)) = N_j_ moles/h* (8)

Δ*G = −(N_i_ + N_j_ ) moles/h* (9)

(10) $$N_{i}=K_{m}(y_{i}-y_{i}*)$$

(11) $$N_{j}=K_{m}(y_{j}-y_{j}*)$$

where *K_m_* is mass transfer coefficient across the porous layer and y_i_* gas concentration of component *i* at the interface of the membrane. According to the presumption made in Equation (6) and Figure 2, the rate determining process among the overall mass transfer process from feed mixture to liquid phase can be the gas flow across the porous layer of membrane. Thus, concentration profile of individual gas component will be developed across the membrane, from concentration *y* in bulk feed to y* at the interface of the layer, and the difference y − y* is a sort of driving force for gas permeation across the gas phase resistance. The mass transfer of permeant across the porous hollow fiber membrane can be depicted by the finite element difference tool below. Consider the absorption mass transfer across the differential section *Dπ* Δ*z* between locations *k* and the *k +* 1 in the grids of the module.

*N*_*i,k+*1_*= G_k_ y_i,k_ − G*_*k+*1_*y*_*i,k*+1_ = *L*_*k+*1_*x*_*i,k+*1_ − *L_k_x_i,k_ = L (x*_*i,k+*1_
*− x_i,k_)* (12)

*N*_*j,k+*1_ = *G_k_* (1 *− y_i,k_*) − *G*_*k+*1_ (1 *− y_i,k+1_)*
*= L (x*_*j,k+*1_
*− x_j,k_)* (13)

N_i,k+1_ = k_m_(y_i,k_ − y_i,k_*)Dπ Δ*z* (14)

N_j,k+1_ = −k_m_(y_i,k_ − y_i,k_*)Dπ Δ*z* (15)

where *D* is the outer diameter of hollow fiber, parameter *k* denotes the k^th^ element volume from the entrance of the module, and the liquid flow rate *L* is presumed to be constant in the membrane contactor process. The relationship between a gas concentration *y* and its equilibrium liquid concentration *x^E^* can follow the Henry law:

*x_i_^E^ = m_i_ P_i_ = m_i_ P y_i_* (16)

*x_j_^E^= m_j_ P_j_ = m_i_ P (*1 *− y_i_)* (17)

where *m_i_* and *m_j_* are the Henry’s constants and *P_i_* and *P_j_* the partial pressures of gas components, *i* and *j*, respectively. Usually, gas permeation rate through the porous layer is identical with the dissolution rate of gas into water because the permeation and the dissolution processes occur in series, and the permeation takes place as fast as the dissolution does. As the liquid flows along the membrane module, a gas component continually dissolves into the liquid stream so that the gas concentration in liquid, *x*, increases with module length and then approaches to the equilibrium concentration, *x^E^*, reducing the dissolution rate of the gas correspondingly. Thus, the permeation rate can be described as a form proportional to the gas concentration difference, *x_i_^E^ − x_i_*, which is a kind of a driving for the permeation of the component through the membrane:

(18) $$N_{i}\propto ({x_{i}}^{E}-x_{i})$$

(19) $$N_{j}\propto ({x_{j}}^{E}-x_{j})$$

Therefore, Equations (14) and (15) can be reformatted into the following equations.

*N*_*i,k+*1_*= R Dπ* Δ*z (x_i_^E^ − x_i_ )* (20)

*N*_*j,k+*1_*= R Dπ* Δ*z (x_j_^E^ − x_j_)* (21)

where *R* is a proportional constant which is the permeability of permeant through a porous membrane. Hagen–Poiseuille equation [17] can express permeability as a function of pore size, porosity, and pore tortuosity. From the analogy between Equations (20) and (21) and Hagen–Poiseuille equation, R-value is postulated to be related to the physical parameters of porous membrane, i.e., pore size, porosity and pore tortuosity. Pore size and porosity will have a positive effect on the R-value. From Equations (12) and (13), equations for gas component concentration dissolved in the liquid can be derived below.

*x*_*i,k+*1_*= x_i,k_ + N*_*i,k+*1_*/L* (22)

*x*_*j,k+*1_*= x_j,k_ + N*_*j,k+*1_*/L* (23)

The initial condition (*k = 0*) for each parameter can be given as follows:

*x*_*i,*0_ = *x*_*i*0_

*x*_*j,*0_*= x*_*j*0_

*y*_*i,*0_*= y*_0_

*L*_0_ = *L*_0_

*G*_0_ = *G*_0_

Combining of Equations (12)–(23) yields the following model equations for feeding into the k^th^ differential element volume in the grids:

*N_i,k_= R Dπ* Δ*z (x_i_^E^ – x*_*i,k −* 1_) (24)

*N_j,k_= R Dπ* Δ*z (x_j_^E^ − x_j,k_)* (25)

(26) $$G_{k}=G_{0}-\;(\sum_{k=1}^{k}N_{i,k}+\sum_{k=1}^{k}N_{j,k})$$

(27) $$x_{i,k}=x_{i,0}+(\sum_{k=1}^{k}N_{i,k}\;)/L$$

(28) $$x_{j,k}=x_{j,0}+(\sum_{k=1}^{k}N_{j,k})/L$$

*y*_*i,k*_*= (G*_*k −* 1_*y*_*k* − 1_ – *N_i,k_)/G_k_* (29)

In chemical industries, components remaining behind after separation of CO_2_ from a gases mixture, such as CH_4_, H_2_, and CO can be valuable products to be obtained from the gassing process in membrane contact. In this sense, the portion of the remaining component harvested from the separation against its initial amount in feed will be one of crucial parameters to evaluate the separation performance. The harvesting parameter is referred to “recovery percent of a component”. In this study, the recovery percent of component *j*, which is a less selectively dissolving component to the liquid, can be defined as the ratio of component *j* flow rate in retentate to its initial flow rate in feed, expressed by the following equation.

*Recovery percent of component j = [G_n_ (*1 *− y_i,n_)]/[G*_0_ (1 *− y*_*i,*0_*)] ∗* 100 (30)

## 3. Experimental

### 3.1. Polypropylene (PP) Hollow Fiber Membrane and Fabrication of the Module

**Figure 4:**
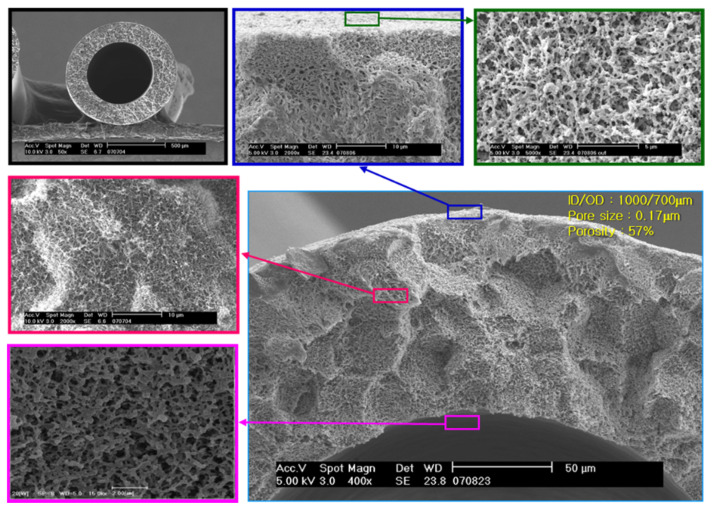
SepraTek’s porous polypropylene hollow fiber membrane fabricated by thermally induced phase separation (TIPS) process.

Water was used as absorbent liquid, to which CO_2_ can be preferentially dissolved against other gases N_2_ or CH_4_. Polypropylene (PP) was chosen as a polymeric material for the membrane contactor membrane because PP is so hydrophobic that it cannot allow the liquid absorbent (water) to penetrate across it. Porous polypropylene hollow fiber membrane was produced by SepraTek (Deajeon, South Korea) proprietary TIPS spinning process without stretching. The PP membrane has a symmetric porous structure as shown in Figure 4, identical in structure from the inner surface through membrane thickness to the outer surface. Membrane module was fabricated by the following procedure.

✓ The PP hollow fibers are woven with a thread or yarn at a regular interval into a hollow fiber membrane mat.
✓ The woven hollow fiber membrane mat is wound around a perforated inner pipe of chlorinated PVC (C-PVC) with a diameter of 16 mm to form a roll of the membrane mat with a diameter of 43 mm. The inner pipe is blocked at the middle of the pipe length
✓ The roll of the membrane mat is put into the module housing of C-PVC with a three-inch inner diameter.
✓ The membrane housing is potted with epoxy resin at its both ends and cut at a location of the potting parts.

**Figure 5:**
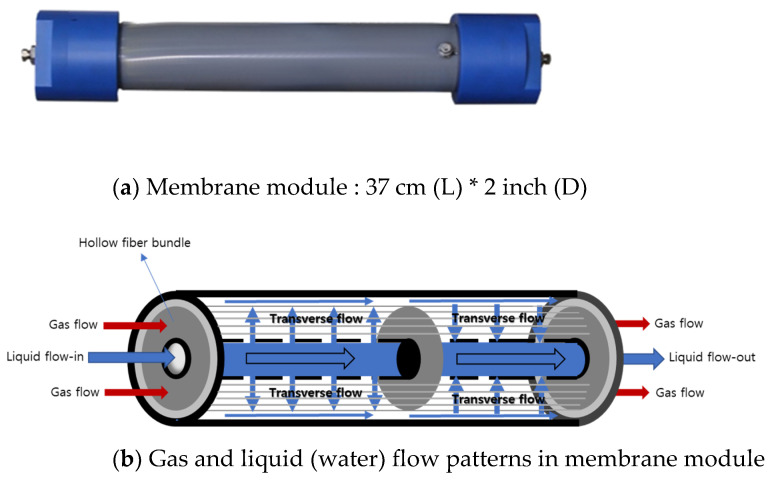
Membrane module used in this study and the schematical representation of membrane module structure and the gas and liquid (water) flows in the membrane module: (**a**) membrane module used in this study and (**b**) gas (lumen side) and liquid flow (shell side) flows in module and module structure.

The purpose to fabricate a bundle of the woven hollow fiber membrane mat is to cause the liquid to flow in transverse mode and to achieve a high degree of mixing of the liquid in flowing in the shell side of membrane module. Blocking of the inner pipe at the middle of the length divides the pipe into two parts: the first half and the second half length parts. When liquid is introduced into the inlet of the first half inner pipe, all the introduced liquid can be induced to flow into the membrane bundle through the holes on the inner pipe because of the blocking of the inner pipe, flowing between hollow fibers in the bundle perpendicularly to the fiber length to cause the transverse flow of the liquid. The liquid flows out from the hollow fiber bundle, flows along the space at the outside of the hollow fiber bundle, flows into the second half length of the bundle in transverse mode, flows out from the bundle into the second half perforated inner pipe, and leaves out the module, as described in Figure 5. Gas mixture flows the lumen side of hollow fiber membrane. The degree of mixing of the liquid in flowing through the membrane bundle can be controlled by the weaving density and pattern of the membrane mat and/or feed flow rate.

### 3.2. Membrane Contactor for Gassing Process

**Figure 6:**
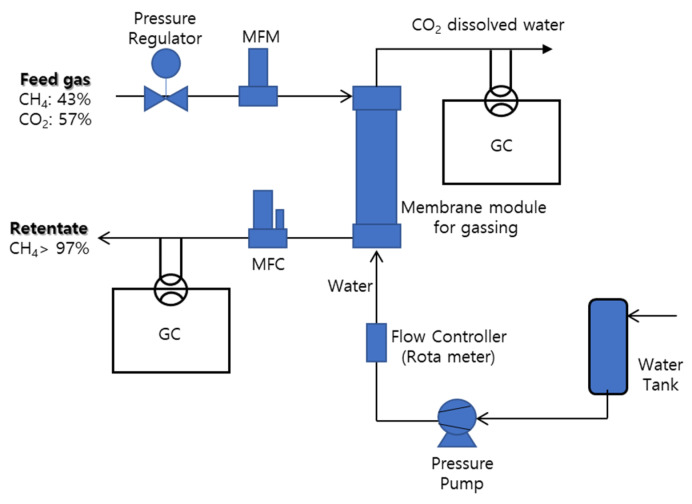
Schematical representation of membrane contactor apparatus for the gassing process of gas mixture (CH_4_/CO_2_).

The membrane contactor apparatus for the gassing process used in this study is illustrated in Figure 6. Two kinds of gas mixtures were employed for membrane contactor process in this study: CH_4_/CO_2_ which is a simulated biogas and N_2_/CO_2_ which is taken as sample gas for typical flue gas. Because CO_2_ can have much higher solubility to water (liquid absorbent) compared to the other two gases as can be depicted in Table 1, membrane contactor process is suitable for the separation of CO_2_ from the gas mixture [1,8,9]. A gas cylinder containing a feed gas mixture was connected to the inlet of the membrane module. CH_4_/CO_2_ with 43/57 in volume percent was used as the feed gas mixture and water was employed as liquid absorbent for CO_2_ in the gassing process. The pressure and the flow rate of gaseous feed were controlled by the pressure regulator and the mass flow controller (MFC, Model 5850E, Brooks Instrument, Japan), respectively. Water was introduced to the shell side of membrane module by the pressure pump (Hydra-cell, Model G-03, USA) under controlling both its flow rate and pressure. The feed gas pressures employed in the gassing process were 5.5, 6.5, and 7.5 kg/cm^2^ and the water stream pressures were maintained 0.5 kg/cm^2^ lower than the employed feed gas pressures, respectively. Feed gas flow rates were 2.866 and 4.286 mol/h. Water flow rates 0.054, 0.060 and 0.069 m^3^/h were used for the feed gas flow of 2.866 mol/h and the water flow rates 0.078, 0.090, and 0.120 m^3^/h were used for the feed gas flow of 4.286 mol/h, respectively. The membrane contactor was carried out for the degassing of the feed gas mixture of CH_4_/CO_2_ at different operating conditions, i.e., various feed pressures, feed flow rates, and water flow rates. The gas flow rates in the feed and the retentate streams were measured by the MFM and MFC installed before and after the membrane module, respectively, and the gas composition of the retentate was directly measured by the GC (Donnam, Model iGC-7200, Korea) which was equipped with a sample injector (six-port valve) actuated by air, a thermal conductivity detector (TCD) and a packed column. The column in the GC was 6 ft long with 1/8 inch inside diameter having a Porapak Q. The GC was also connected with the computer by the 21-bit interfacial module for data acquisition and the determined concentrations could be displayed at the computer monitor.

## 4. Results and Discussion

### 4.1. Verification of the Simulation Model Validity

The simulation equations were solved by a numerical method of Finite Element Method (FEM) and the program coding was done by Turbo Pascal (Borland, Austin, TX, USA). To verify the established simulation model, the simulated values were compared with the experimental values obtained from the real degassing process of CH_4_/CO_2_ mixture using the PP hollow fiber membrane module. All of parameters which should be known to simulate the model are *D,* Δ*z*, *G*_0_, *L*_0_, *y*_*i*,0_, *x*_*i*,0_, *x*_*j*,0_, *R*, *m_i_*, and *m_j_*. Among them, the parameters, *D*, Δ*z*, *G*_0_, *L*_0_, *y*_*i*,0_, *x*_*i*,0_, *x*_*j*,0_ were given as operation parameter in the process, and *m_i_* and *m_j_* were obtained from literatures and publications [18] and presented in Table 1. Only R needs to be determined, which can be obtained by a semi-empirical method described below.

Total and CH_4_ component flow rates in retentate were obtained at different feed pressures, feed flow rates, and liquid flow rates, respectively, from the gassing process of CH_4_/CO_2_ mixture through the PP hollow fiber membrane module. The parameter R (hereafter called as R-value) was determined by fitting the experimental data into the model equations. The determined R-value was 0.155 for the PP hollow fiber membrane with 0.58% porosity, 0.18 mm pore size (by bubble point measurement) and 1000/700 μm for OD/ID.

**Figure 7:**
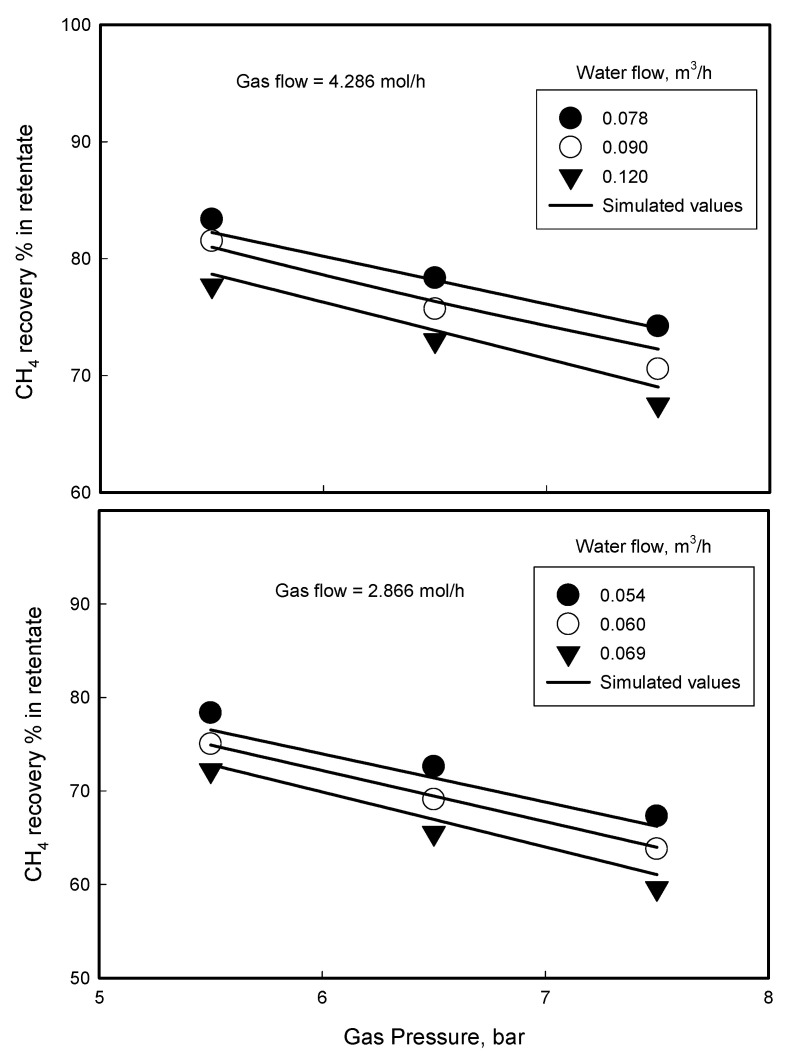
A comparison of simulated CH_4_ recovery percent in retentate to the real experimental value in membrane contactor separation of CH_4_/CO_2_ mixture at different feed gas flow rates and gas/liquid flow rate ratios.

The simulation for the gassing process of CH_4_/CO_2_ mixture was carried out with the help of the model equation using the determined R-value and the same operation conditions as the ones used in the experiment, and a comparison of the simulated values to the experimental data was made and illustrated in Table 2 and Table 3 and Figure 7. It is found from the comparison that the simulation has a good agreement with the experimental data. From this finding, it can be seen that the established simulation model is of practical value to predict and analyze the membrane contactor behavior.

It is observed from the gassing process that the permeation rate of CH_4_ gas into water increases with increasing the feed gas pressure and the CH_4_ recovery percent in retentate decreases, correspondingly. According to Henry’s law in Equations (17) and (16), as feed gas pressure increases, more gas molecules dissolve to water, and thereby permeation rate of the gas will increase, losing more of the gas into water and resulting in lower gas recovery percent in retentate. Finding of lower CH_4_ recovery percent or higher CH_4_ permeation rate for higher water flow rate is attributed to the gas concentration *x* in water reduced by the dilution of the gas component with larger water flow, which increases the driving force *x^E^ − x* for the permeation in Equation (21).

### 4.2. Simulation of Separation of Gas Mixture in Membrane Contactor Process

The membrane contactor process can be applicable to separate acidic gases, such as CO_2_, sulfur oxide (SOx) and nitrogen oxide (NOx) in flue gas because their solubilities in water are higher than the other gases, so the acidic gases could be preferentially dissolved to water and separated effectively from the flue gas. For the separation of CO2 from flue gas, CO_2_/N_2_ mixtures have been frequently used as a typical example of flue gas [7,8,9]. In this study, a CO_2_/N_2_ mixture was employed to investigate the separation behavior of CO_2_ from the mixture in membrane contactor with help of the verified simulation model, and the simulation for membrane contactor was carried out for a parametric study on the separation of CO_2_ through the gassing process. 

**Figure 8:**
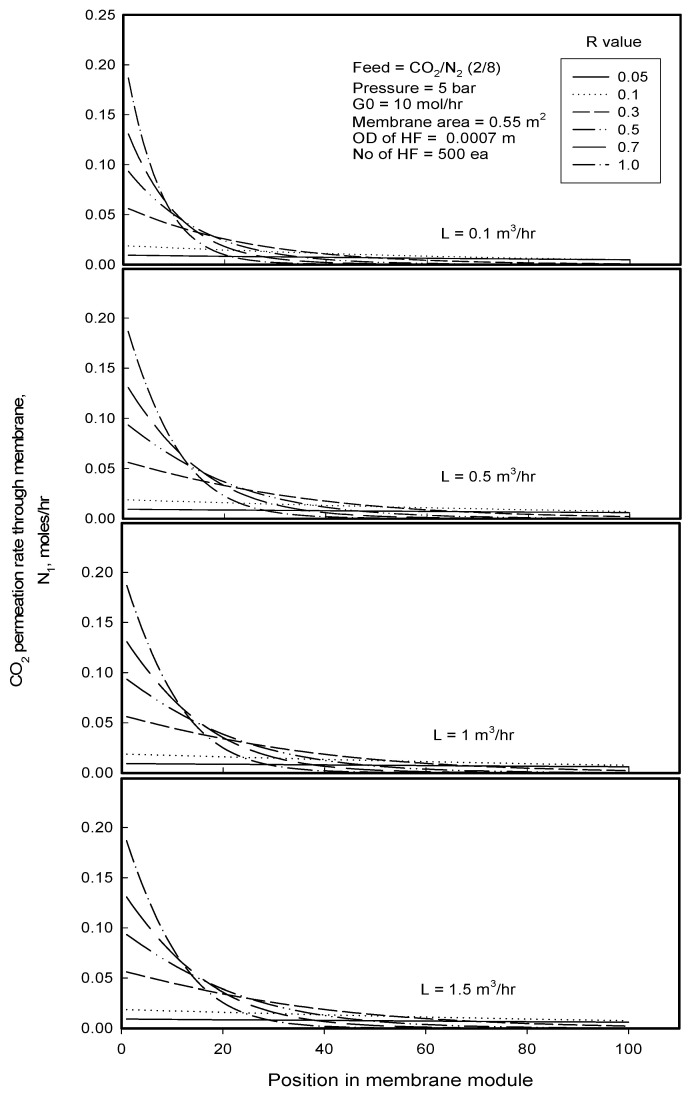
CO_2_ permeation rate simulated with the position of membrane module in gassing process for the separation of CO_2_/N_2_ mixture at different liquid flow rates and R-values.

Figure 8 shows CO_2_ permeation rate simulated with the position of membrane module length in the gassing process. At the position zero which is designated to the inlet of membrane module, CO_2_ permeation rate increases through the membrane with increasing the R-value. As explained previously, the R-value has something to do with mass transfer characteristic dependent on the structure of porous membrane, being larger in value for more porous membrane. Therefore, it is clearly understandable that the permeation rate through membrane of larger pores or larger porosity is higher. With flowing along the membrane module, the permeation rate decreases and then levels off to a value near zero. It is because CO_2_ permeates preferentially through the porous membrane and dissolves into the water, and thereby, CO_2_ can become depleted more and more in feed stream with the position of module length to cause decrease in the permeation rate and to be leveled off to zero. It is observed that the permeation rate decreases more rapidly and is leveled off faster when the R-value is higher. The rapid decline in the permeation rate can be explained by (1) permeating faster a permeant through a membrane of larger mass transfer coefficient, (2) depleting faster the permeant in the feed stream and then (3) lowering more the permeation rate of the depleted permeant. The level-off of permeation rate with the position of module length might be attributable to CO_2_ concentration *x* in the liquid increasing to approach its equilibrium concentration *x^E^* and thereby driving force for the permeation reducing to zero as expressed in Equations (18) and (19), which will be discussed in the change of CO_2_ concentration in liquid stream with the position of module length in gassing process later. At the module inlet, it is also found that the gas permeation rate through membrane with a given R-value is constant regardless liquid flow rate because the gas concentration *x* in the liquid stream is supposed to be zero at the module inlet so that the driving force (*x^E^* − *x* = *x^E^*) for permeation would be the same, resulting the same permeation rate for the same driving force and R-value, according to Equations (20) and (21).

**Figure 9:**
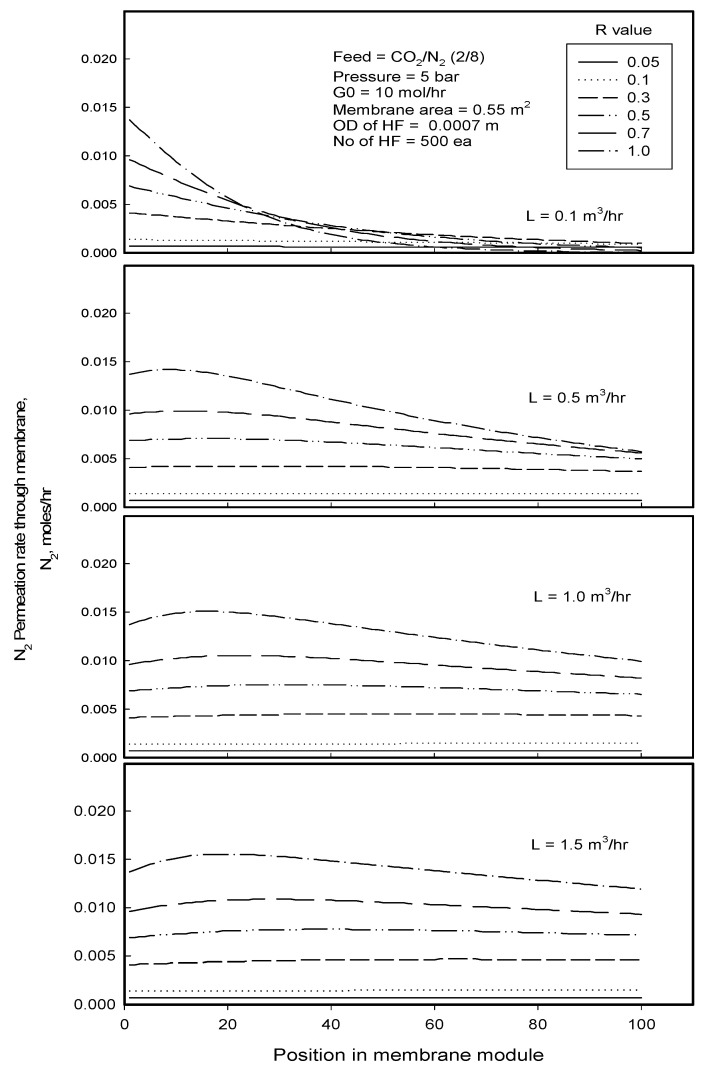
N_2_ permeation rate simulated with the position of membrane module in gassing process for the separation of CO_2_/N_2_ mixture at different liquid flow rates and R-values.

Figure 9 presents N_2_ permeation rate simulated with the position of module length in gassing process for the separation of CO_2_/N_2_ mixture at different liquid flow rates and R-values. Like Figure 8, N_2_ permeation rate grows higher through the membrane with higher R-value at the membrane module inlet and levels off faster to a value for the same reason as in Figure 8, especially when the liquid flow rate is low, 0.1 m^3^/h. As the liquid flow rate is increased, N_2_ permeation rate increases as much. 

The increase in gas permeation rate was explained [1,8] by increase in the mass transfer of the permeant with increasing water flow rate through reducing boundary layer thickness, channeling phenomenon, and bypassing effect in water stream. However, it is not the case for the module employed in this study. As explained previously, the membrane module employed in this study was designed for transverse flow of water and maximizing the mixing of water stream in the module, so the water flow rate would scarcely affect the boundary layer thickness, channeling, and the bypass effect. Even if water flow rate has some effect on the mass transfer of gas component, it would not be remarkable. The observation can be attributed to the dilution of N_2_ by large water flow: CO_2_, which is preferentially dissolved in the liquid, is dissolved more in water than N_2_, which makes N_2_ more concentrated in the feed stream and makes N_2_ equilibrium concentration *x^E^* increase correspondingly in the liquid, while component N_2_ can be diluted at the same time in the liquid due to large liquid flow, and its concentration x in the liquid becomes reduced as much (the dilution effect of water flow) so that the driving force for N_2_ permeation or dissolution, *x^E^ − x* increases as much, increasing the permeation rate with liquid flow rate. It is interesting to observe that the permeation rate increases and then decreases with the position of module length, showing a convex shape of a curve upward which is more remarkable for higher R-value. It can be explained by competing effects of the increase in N_2_ concentration in both the feed gas stream and the liquid stream on the driving force, affecting the permeation in the opposite way: concentrating N_2_ in the feed stream is positively affected by increasing *x^E^*, while concentrating of N_2_ in the liquid stream negatively affects the N_2_ permeation through decreasing the driving force with the position of module length. Looking at the tendency to change the permeation rate of CO_2_ in flowing along the module length in Figure 8, the permeation of CO_2_ takes place significantly at the inlet of the membrane module and the permeation rate lowers and levels off with the position of module length. From these findings, it can be postulated that the concentrating effect of N_2_ in the feed stream might be more dominant rather than the concentrating effect in the liquid stream in the incipient stage of the degassing process due to the rapid depletion of CO_2_ component in the feed stream, but as flowing along the module, the permeation of CO_2_ is diminished rapidly and concentration of N_2_ in the feed side is reduced as much, while the concentration of N_2_ in the liquid side keeps increasing by continually dissolving in the liquid as long as N_2_ concentration in the feed stream maintains lower than the value in equilibrium with the concentration in the liquid as will be shown later, so that the concentrating effect of N_2_ in the liquid side would be more predominant on the N_2_ permeation.

**Figure 10:**
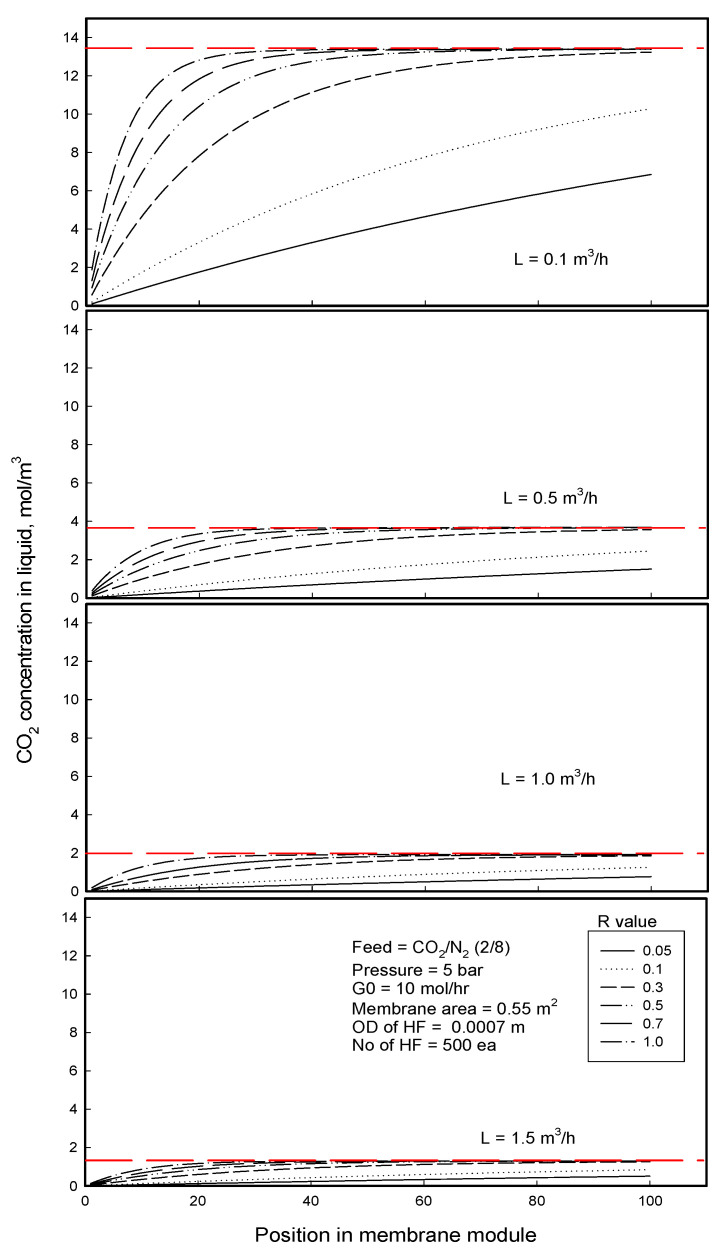
CO_2_ concentration in liquid stream with the position of module length in gassing process for the separation of CO_2_/N_2_ mixture: red broken line = *x^E^*, equilibrium CO_2_ concentration in the liquid stream.

**Figure 11:**
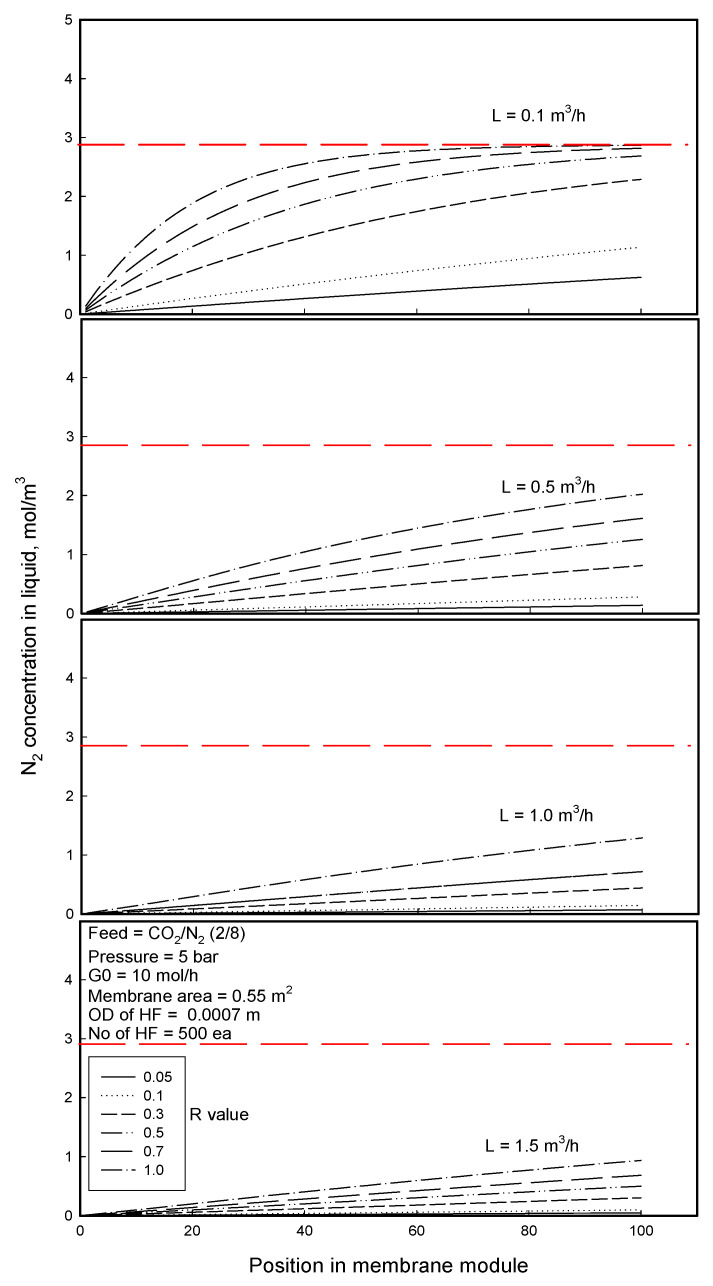
N_2_ concentration in liquid stream with the position of module length in gassing process for the separation of CO_2_/N_2_ mixture: red broken line = *x^E^*, equilibrium N_2_ concentration in the liquid stream.

Figure 10 and Figure 11 show CO_2_ and N_2_ concentrations in the liquid stream with the position of module length in gassing process for the separation of CO_2_/N_2_ mixture. On the whole, the concentration of CO_2_ having higher Henry’s constant is higher than N_2_ concentration in the liquid. Regardless of liquid low rate, CO_2_ concentration increases and levels off faster as approaching its equilibrium concentration in the liquid as flowing along the module when the R-value of membrane is higher. The higher R-value induces higher mass transfer of a permeant through the membrane, and the permeant molecules permeate and dissolve faster enough to reach the equilibrium concentration in the liquid before leaving the membrane module. On the other hand, when R-value is too small, because the mass transfer of the permeant is too small, the permeant molecules permeate and dissolve too slowly to reach the equilibrium point but the concentration in the liquid increases continually with the position of module length, increasing less as approaching the equilibrium concentration in the liquid. As for the dissolution behavior of N_2_ component in the liquid, the concentration increases in a more curved shape for higher R-value and/or lower liquid flow rate, which has already been explained in terms of the opposite effects of the increase in N_2_ concentration in both the feed gas and the liquid streams on the driving force. As the concentration approaches its equilibrium point, it increases less and less and levels off to the equilibrium value, being associated with decreasing the driving force *x^E^ − x*. The broken lines in Figure 10 and Figure 11 denote the equilibrium concentrations of the liquid with different flow rates, respectively. The equilibrium concentration of CO_2_ is observed as a function of liquid flow rate regardless of R-value, while the N_2_ equilibrium concentration is constant in the range of liquid flow rates employed in this study. A good separation should show a high permeation rate of target component (CO_2_) and a low permeation rate of the other component (N_2_) (in order to minimize losses) together with low energy consumption. From this viewpoint, although R-value and water flow rate affect the permeation rates of both components in the same way, as water flow rate or R-value is higher, the permeation rate ratio of CO_2_ to N_2_ is decreased, indicating that the N_2_ loses more by dissolution to water. Thus, Optimization of the operation parameters will be discussed, compromising between the N_2_ recovery percent and separation efficiency.

**Figure 12:**
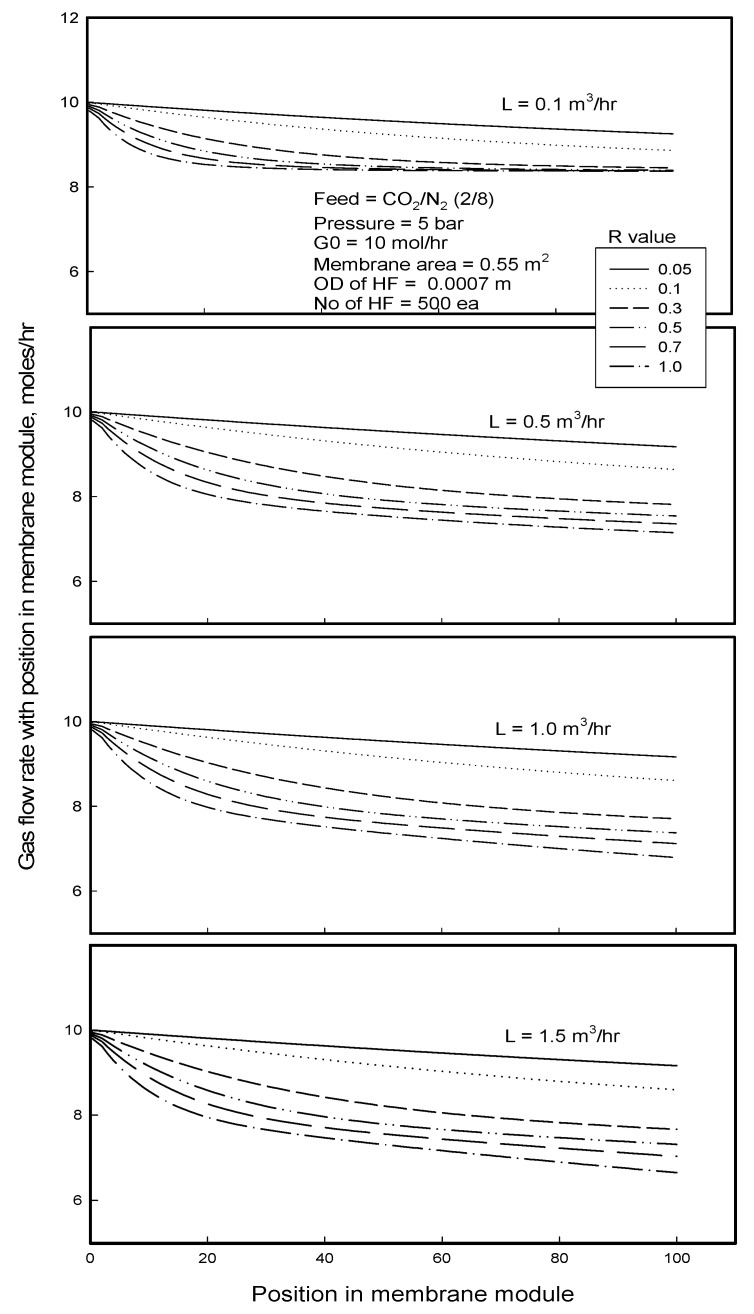
Gas flow rate in feed stream with the position of membrane module length in gassing process for the separation of CO_2_/N_2_ mixture.

At a given liquid flow rate, feed gas flow rate along the module length is reduced as much as the sum of the permeation rates of gas components through the membrane (Figure 12). Decreasing the gas flow rate is more remarkable for the membrane with higher R-value due to higher permeation rate of gas component. When the liquid flows faster in the module, the liquid allows more gas molecules to be dissolved in it, expediting to permeate them especially through membrane with larger R-value while reducing feed gas flow rate as much. It is interesting to find that the feed gas flow rate in the module with high R-value decreases and then levels off with the position of module length when the liquid flow rate is low enough (0.1 m^3^/h). However, the feed gas flow rate keeps on decreasing with the position of module length when water flow rate is 0.5 m^3^/h or higher, decreasing more significantly as the liquid flow rate increases. It can be explained by the dissolved gas amount with liquid amount as mentioned in Figure 11. When liquid flow is slow enough, the dissolution of gas permeant to the liquid tends to be easily saturated before leaving out the module, especially for a gas component with a high solubility, like CO_2_, while when the liquid flow is faster than a critical value, the gas dissolution is not saturated due to large volume of the liquid passed through the module, so the feed gas flow rate is decreased as flowing in the module, mainly due to continually dissolving N_2_ into the liquid stream.

**Figure 13:**
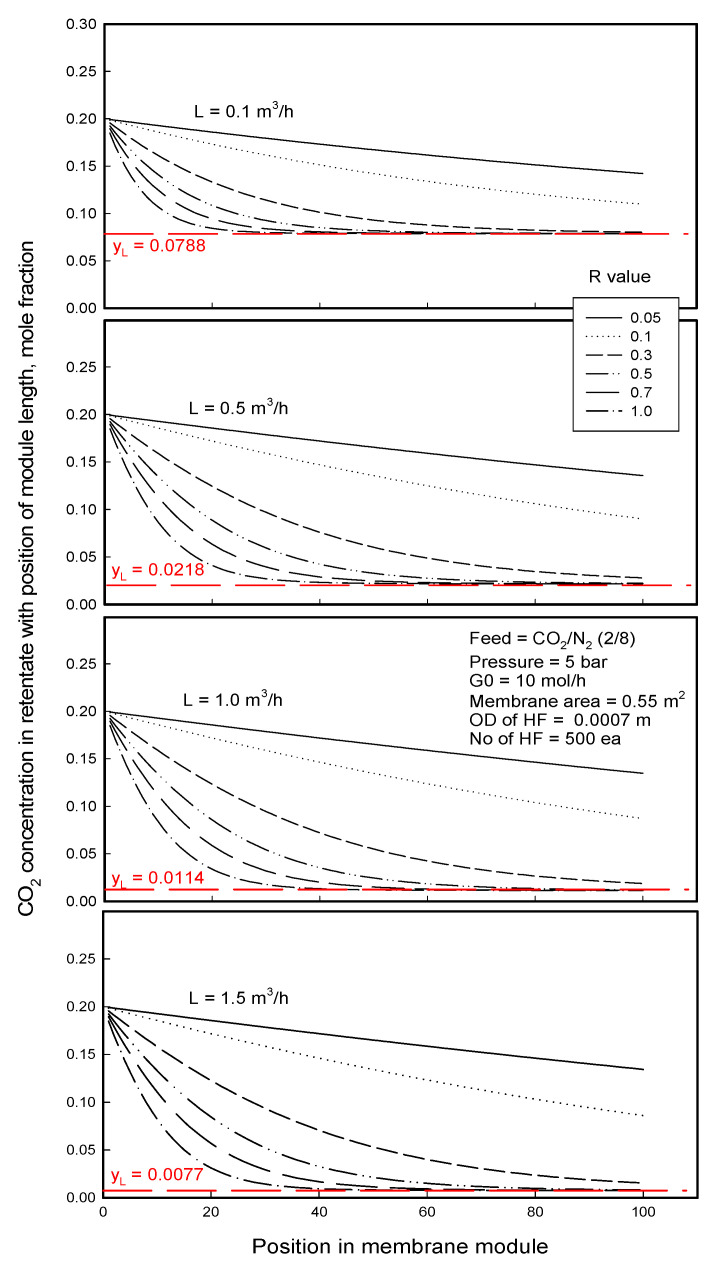
CO_2_ concentration in feed flow module with position of porous polypropylene membrane in membrane contactor separation of CO_2_/N_2_ mixture, and *y_L_* = CO_2_ concentration in feed stream adjacent to the interface, in equilibrium with the saturated concentration of CO_2_ *x_i_^E^* in the liquid stream.

Figure 13 shows a change in CO_2_ concentration in feed stream with the position of module length at various liquid flow rates through the membranes of different R-values. The CO_2_ concentration change in the feed gas stream can be explained in association with the dissolution behavior of CO_2_ into the liquid as shown in Figure 10. The CO_2_ concentration decreases in the feed stream with the position of module length, which is attributed to selectively permeating and dissolving into the liquid stream. The concentration in the feed stream decreases less and less and levels off to a certain value as CO_2_ concentration in liquid approaches its equilibrium concentration in the liquid stream, especially when the R-value of membrane is large, above 0.3. The level-off values *y_L_* denoted as the broken line in Figure 13 are the concentration in the feed gas in equilibrium with *x^E^* in the liquid shown in Figure 10. Like *x^E^*, the concentration *y_L_* is constant with R-value in each liquid flow rate and is a function of the liquid flow rate for the same reason. CO_2_ concentration in the retentate is decreased with increasing R-value until reaching the equilibrium value, *y_L_*, and then is constant with R-value at a given water flow rate. Retentate with low CO_2_ concentration can be obtained with higher water flow rate. The composition of retentate would have something to do with separation efficiency. Therefore, water flow rate or membrane porous structure can be given from the simulation to attain the target purity of a gas component in retentate.

**Figure 14:**
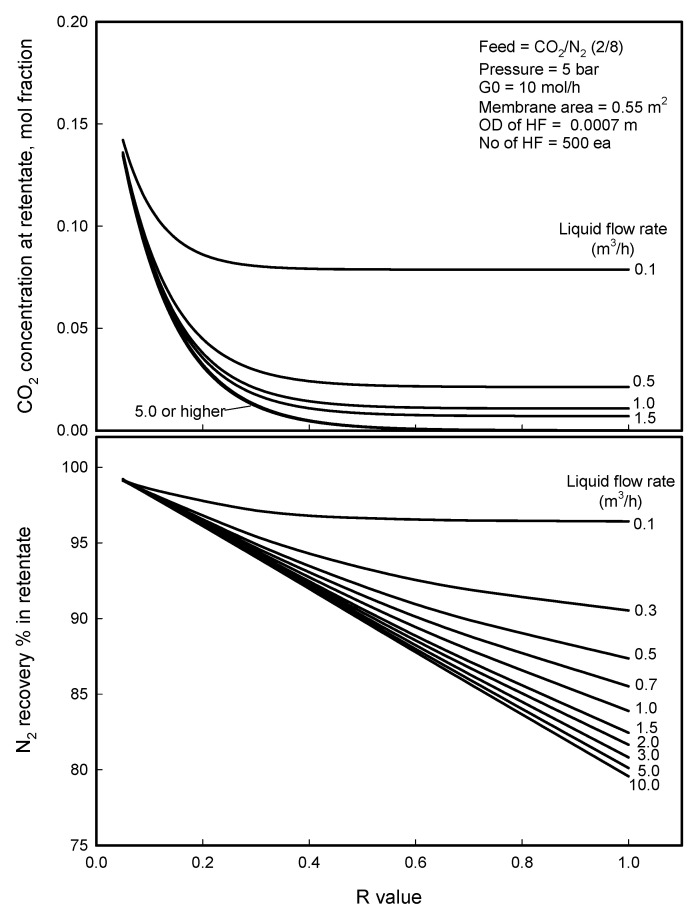
CO_2_ concentration and N_2_ recovery percent of retentate stream with R-value in the gassing of N_2_/CO_2_ mixture in membrane contactor process.

As mentioned above, N_2_ recovery percent is the portion of residual N_2_ in the retentate stream excluding the loss of N_2_ through the permeation and dissolution into the liquid stream. More permeation and dissolution of N_2_ component into the liquid yields lower N_2_ recovery percent, so recovery percent can be used to evaluate the permeation amount of the component in a separation. The composition of retentate would have something to do with separation efficiency. An ideal separation should have a maximum N_2_ recovery percent in retentate (minimum permeation of N_2_) and a minimum CO_2_ concentration in retentate (maximum permeation of CO_2_). Figure 14 presents the plots of N_2_ recovery percent and CO_2_ concentration in retentate stream against R-value at different liquid flow rate, respectively. CO_2_ concentration in retentate decreases with increasing R-value until about 0.3 and then levels off to a value which decreases with water flow rate. Very pure N_2_ (> 0.99 mol fraction) can be harvested from retentate when water flow rate is larger than 1.5 m_3_/h. N_2_ recovery percent tends to reduce with R-value owning to N_2_ loss via large permeation of N_2_ through membrane with large mass transfer coefficient. When the R-value is 0.05 or smaller, N_2_ recovery is almost constant, near 100%, regardless of liquid flow rate. With increasing of R-value, N_2_ recovery percent decreases from the constant value, decreasing more remarkably for higher liquid flow rate. When the liquid flow rate is 0.1 m^3^/h or lower, N_2_ concentrations in the liquid stream are very close to the equilibrium concentration at the module outlet for R-value of higher than 0.3, as can be seen in Figure 11, causing the permeation rate of N_2_ to be near zero (Figure 9) because of almost zero driving force *x^E^–x* for the permeation of N_2_. That is why the N_2_ recovery percent curve is flatter in shape with R-value for the liquid flow rate. As increasing the liquid flow rate, the recovery percent decreases with R-value in less curvature with asymptotically increasing slope and decreases linearly with a slope when the liquid flow rate is 10 m^3^/h or higher. For maximum separation performance in membrane contactor, the parameters should be optimized to obtain a low CO_2_ concentration, a high N_2_ recovery percent in retentate and low energy consumption (low water flow rate): a good separation performance of CO_2_ concentration of 0.01 mol. fraction and N_2_ recovery percent of 95 in retentate is likely to be obtained under R-value of 0.32 and water flow rate of 1.5 m^3^/h from Figure 14.

**Figure 15:**
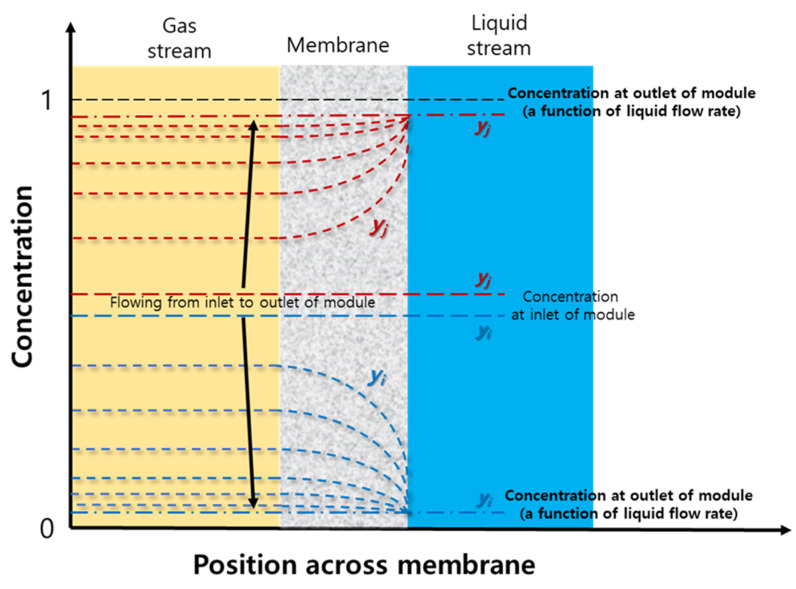
Schematical representation of the concentration profile of individual gas component developed in feed stream and across membrane in the gassing process for the separation of a mixture of components *i/j*. (*i*: preferentially dissolving component to the liquid).

From these observations, it can be found that when liquid flow rate is small enough, the liquid stream will be saturated easily with the dissolved gas due to long dwelling of the liquid in the module to such an extent that the equilibrium point could be reached quickly, and the permeation and dissolution of the gas will stop before leaving the module. In this case, the dissolution of the gas in the liquid will determine the permeation rate. When liquid flow rate is sufficiently large, the liquid stream will hardly be saturated with the gas because of short dwelling of the liquid in the module. The recovery percent has a liner relationship with R-value no matter how fast the liquid flows in the module, indicating that the permeation is affected mainly by the permeation through the membrane rather than the dissolution into the liquid, which means the permeation through membrane would be a rate determining step in the gassing process. From the result of the simulation of the gassing process for the separation of components *i/j* (CO_2_/N_2_) mixture, the concentration profile of individual component can be envisaged to be developed across the membrane in flowing along the membrane module as described in Figure 15. With the feed stream flowing to the module outlet, N_2_ concentration at the interface with the liquid phase increases while CO_2_ concentration decreases owing to the preferential dissolution of CO_2_ to the liquid so the developed concentration profiles of the gas components could make N_2_ permeation more facilitated while the CO_2_ permeation more depressed. N_2_ recovery percent will be reduced as much as N_2_ permeation through membrane. From this viewpoint, the facilitation of the N_2_ permeation should be alleviated, and the depression of CO_2_ permeation should be mitigated as much as possible by optimizing the concentration profiles developed in across the membrane to achieve maximized separation of N_2_/CO_2_. 

It can be drawn from the analysis above that liquid flow rate and R-value affect the separation performance in opposite way; liquid flow rate has a negative effect on separation efficiency, i.e., N_2_ recovery but a positive effect on the separation of CO_2_, while R-value affects the separation performance in the other way. Thus, the practical simulation model developed in this study can be used as a tool to find the parameters, liquid flow rate and R-value to maximize the separation performance in terms of N_2_ recovery percent and CO_2_ permeation.

## 5. Conclusions

A practical simulation model for separation of gas mixture in membrane contactor process has been developed, based on mass balance and mass transport equation and equilibrium equation between gas and liquid phases across porous membrane. In order to determine a practical value of the parameter, R-value, which is related to the permeability of gas permeant across porous membrane, has been determined by fitting a numerical solution of the model equation to experimental data obtained in real membrane contactor process through porous PP (polypropylene) hollow fiber membrane module. The separation of CH_4_/CO_2_ mixture by membrane contactor process has been simulated by the established simulation model employing the R-value determined from the phenomenological approach. Comparisons were made between the simulated and the experimental values to verify the established simulation model. It is confirmed that the simulation values are in a good agreement between the experimental values, meaning that the determination methodology for R-value would be practical.

Through the parametric study on the gassing process for the separation of CO_2_ from the N_2_/CO_2_ mixture with help of the established simulation model, the effects of membrane structure, liquid flow rate and feed gas pressure have been investigated on the separation performance in the gassing process of N_2_/CO_2_ mixture in membrane contactor. It was found especially that the dissolution of gas component to the liquid will determine the permeation rate when liquid flow rate is small enough, while the permeation through membrane would be a rate determining step in the gassing process when liquid flow rate is sufficiently large.

To achieve effective separation of N_2_/CO_2_, the permeation and dissolution of N_2_ component into the liquid stream should be depressed while the permeation and dissolution of CO_2_ component should be expedited as much as possible. It could be drawn from the analysis of the result of the simulation that liquid flow rate has a negative effect on N_2_ recovery percent in retentate but a positive effect on the separation of CO_2_, while R-value affects the separation performance in the other way. Thus, the liquid flow rate and the R-value should be compromised to maximize the separation performance in terms of N_2_ recovery percent and CO_2_ permeation rate. It is confirmed that the simulation model developed in this study can be used as a tool to optimize the parameters, i.e., feed gas pressure, liquid flow rate and R-value to maximize the separation performance.

## Figures and Tables

**Table 1:** Henry constants of gases in water published in a literature [18].

Gas	Henry Constant, mol/m^3^-atm	Remarks
CO_2_	34.00	25 °C
N_2_	0.625	25 °C
CH_4_	1.420	20 °C

**Table 2:** A comparison of simulated retentate gas flow rate to experimental value in purification of CH_4_ by membrane contactor at various feed pressures and flow rates through porous PP hollow fiber membrane with OD/ID = 1000/700 μm and a porosity of 58%, number of HF in membrane module = 700, effective membrane length = 29 cm, membrane area = 0.64 m^2^ and the determined R-value = 0.155. *( )/( ): *experimental value/simulated value.

Gas Pressure, kg/cm^2^	Feed, Mole/h		Retentate, Mole/h		Remarks
	Total Flow mol/h	CH_4_ Flow mol/h	Total Flow mol/h	CH_4_ Flow mol/h	
5.5	2.866	1.232	* 1.021/1.106	* 0.965/0.943	G_0_ = 2.866 mol/h L_0_ = 0.054 m^3^/h
6.5	2.866	1.232	0.940/1.008	0.895/0.880	
7.5	2.866	1.232	0.870/0.919	0.829/0.816	
5.5	2.866	1.232	0.973/1.067	0.924/0.923	G_0_ = 2.866 mol/h L_0_ = 0.060 m^3^/h
6.5	2.866	1.232	0.891/0.968	0.851/0.856	
7.5	2.866	1.232	0.819/0.878	0.787/0.788	
5.5	2.866	1.232	0.935/1.018	0.890/0.897	G_0_ = 2.866 mol/h L_0_ = 0.069 m^3^/h
6.5	2.866	1.232	0.843/0.919	0.807/0.825	
7.5	2.866	1.232	0.765/0.827	0.707/0.753	
5.5	4.286	1.843	1.642/1.789	1.536/1.515	G_0_ = 4.286 mol/h L_0_ = 0.078 m^3^/h
6.5	4.286	1.843	1.535/1.659	1.444/1.440	
7.5	4.286	1.843	1.438/1.543	1.368/1.364	
5.5	4.286	1.843	1.609/1.726	1.503/1.492	G_0_ = 4.286 mol/h L_0_ = 0.090 m^3^/h
6.5	4.286	1.843	1.476/1.598	1.395/1.412	
7.5	4.286	1.843	1.369/1.483	1.300/1.332	
5.5	4.286	1.843	1.525/1.620	1.432/1.450	G_0_ = 4.286 mol/h L_0_ = 0.120 m^3^/h
6.5	4.286	1.843	1.401/1.495	1.328/1.361	
7.5	4.286	1.843	1.302/1.380	1.214/1.272	

**Table 3:** Comparison of calculated CH_4_ recovery percent to real values through porous polypropylene hollow fiber membrane with OD/ID = 1000/700 mm, number of HF = 700, Effective membrane length = 29 cm, determined R value = 0.155, *( )/( ): *experimental value/simulated value.

Gas Pressure, kg/cm^2^	G_0_ = 4.286 mol/h L_0_ = 0.078 m^3^/h	G_0_ = 4.286 mol/h L_0_ = 0.090 m^3^/h	G_0_ = 4.286 mol/h L_0_ = 0.120 m^3^/h
5.5	* 83.35/82.24	* 81.50/80.98	* 77.70/78.67
6.5	78.34/8.16	75.68/76.34	73.00/73.86
7.5	74.21/74.05	70.54/72.26	67.50/69.01
Gas Pressure, kg/cm^2^	G = 2.866 mol/h L = 0.054 m^3^/h	G = 2.866 mol/h L = 0.060 m^3^/h	G = 2.866 mol/h L = 0.069 m^3^/h
5.5	78.35/76.53	75.02/74.91	72.20/72.82
6.5	72.60/71.40	69.09/69.46	65.50/66.96
7.5	67.30/66.23	63.79/63.98	59.60/61.06
